# Structural Insights into Escherichia coli Fructose-1-Phosphate Kinase Reveal Evolutionary Divergence within the PfkB Family

**DOI:** 10.1063/4.0001173

**Published:** 2025-10-27

**Authors:** Soyoung Bae, Katie M Satko, Dean R Tolan

**Affiliations:** Boston University, Boston, MA, USA

## Abstract

Fructose-1-phosphate kinase (FruK) catalyzes the ATP-dependent phosphorylation of fructose 1-phosphate to fructose-1,6-bisphosphate in many bacterial species, yet this enzyme is notably absent in eukaryotes (Ferenci & Kornberg, 1973; Hanson & Anderson, 1968; Buschmeier et al., 1985). FruK belongs to the phosphofructokinase B (PfkB) family of carbohydrate kinases (Pfam PF00294), which broadly functions to transfer a phosphate group from ATP to the hydroxymethyl or nitrogen moiety of sugars and related substrates such as adenosine, inosine, or modified sugars (Sigrell et al., 1998; Titgemeyer et al., 1994; Park & Gupta, 2008). Despite the functional diversity of PfkB enzymes, they share a conserved structural core characterized by a central eight-stranded αβα sandwich, flanking helices, and often a β-sheet domain acting as a thumb (Park & Gupta, 2008). The family is conserved across all phyla, with human homologs including ribose kinase, adenosine kinase, and ketohexokinase (KHK). Here, we present a 2.10 Å-resolution crystal structure of Escherichia coli FruK in complex with fructose-1-phosphate, accompanied by a comparative analysis with representative PfkB family members. This structural comparison reveals conserved motifs essential for catalytic function as well as distinctive features that may account for FruK’s substrate specificity. Together, these findings deepen our understanding of structure–function relationships within the PfkB family and illuminate evolutionary adaptations unique to FruK.

